# Dorsal Onlay Oral Mucosa Graft Urethroplasty: A Case Report and Review of Literature

**DOI:** 10.1155/2020/8822007

**Published:** 2020-10-05

**Authors:** Kwaku Addai Arhin Appiah, Christian Kofi Gyasi-Sarpong, Edwin M. T. Yenli, Patrick Opoku Manu Maison, Charles Kwame Adofo, George Amoah, Roland Azorliade, Dominic Annor Mintah, Augustina Badu-Peprah

**Affiliations:** ^1^Department of Surgery, Komfo Anokye Teaching Hospital, Kumasi, Ghana; ^2^Department of Surgery, KSMD, College of Health Sciences, KNUST, Kumasi, Ghana; ^3^Kings Medical Consult (Specialist Urology Clinic), Kumasi, Ghana; ^4^University for Development Studies-SMHS, Department of Surgery, Urology Unit, Tamale, Ghana; ^5^Department of Surgery, School of Medical Sciences, UCC, Cape Coast, Ghana; ^6^Seventh Day Adventist Hospital, Dominase, Ghana; ^7^Department of Radiology, Komfo Anokye Teaching Hospital, Kumasi, Ghana; ^8^Department of Radiology, KSMD, College of Health Sciences, KNUST, Kumasi, Ghana

## Abstract

The use of buccal mucosa grafts in urethral reconstruction for complex anterior urethral strictures has gained popularity over the years with very good outcomes reported in literature. We report on the successful repair of a complex anterior urethral stricture in a 14-year-old boy following catheterization using this method at the Komfo Anokye Teaching Hospital. The aim is to describe the method of dorsal onlay oral mucosa graft urethroplasty and to review the literature.

## 1. Introduction

Urethral strictures in children are uncommon. They may present as lower urinary tract symptoms or acute urine retention [1]. The causes could be congenital, iatrogenic, posthypospadias repair, or traumatic [2, 3]. The diagnosis involves a detailed history, physical examination, and appropriate radiological and endoscopic investigations [4].

Retrograde urethrography (RUG) with or without micturating cystourethrogram (MCUG) is the gold standard confirmatory test. In some situations, urethroscopy may be required for confirmation and treatment. Penile and long bulbar urethral strictures are generally regarded as complex strictures. This is because they do not lend themselves to simple excision and primary anastomosis [5].

They require tissue transfer in the form of grafts, flaps, or staged repair. Since the 1990s, the use of the buccal mucosal grafts to repair such complex anterior strictures has gained enormous popularity and acceptance due to its reproducibility and durable long term success rates of 85% and beyond [6–8].

Of significant debate is whether to place the graft ventrally or dorsally on the urethra. The dorsal approach seems cumbersome but the outcome is definitely better due to the mechanical support offered to the graft by the ventral aspect of the corporeal bodies [9–11].

We report on the successful application of the use of the oral mucosa graft dorsal onlay urethroplasty technique as described by Guido Barbagli [12] in a 14-year-old boy with a 7 cm Peno-bulbar urethral stricture at the Komfo Anokye Teaching Hospital (KATH). The objective is to highlight the technique of dorsal onlay oral mucosa graft urethroplasty and to review the literature.

## 2. Case Report

A 14-year-old boy had laparotomy for a mesenteric cyst 7 years ago during which he had a transurethral bladder catheter passed. Few weeks after discharge from the hospital, he began to experience difficulty in passing urine. It started as straining at urination with a poor stream which worsened over time. Over the cause of the past 7 years, he has had multiple dilatations which did not resolve the problem until a suprapubic catheter was passed for him a year ago and referred to see the urologist at KATH.

On examination, he had normal growth for his age. He had a two-way suprapubic catheter in situ with indurations along the penile and bulbar urethra.

**Figure 1:**
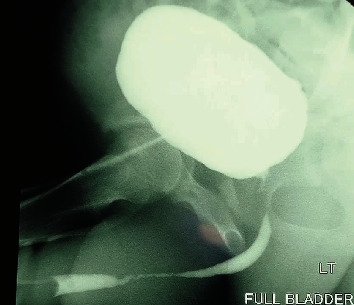
RUG in the left oblique view showing a 7 cm long partial occlusion of the penile and bulbar urethra.

A retrograde urethrogram demonstrated a partial narrowing of the penile and bulbar urethra (Figure 1).

### 2.1. Preoperative Preparation

A routine urine examination was done which revealed leukocytosis for which reason a urine culture was requested that isolated Klebsiella species sensitive to only amikacin.

A one-week course of parenteral amikacin was given after which a repeat urine culture was negative.

Other tests done included a full blood count and kidney function tests which were all within normal ranges. Thus, patient was scheduled for urethroplasty.

Patient positioning was the standard lithotomy with padding of the pressure points with administration of general anaesthesia with oropharyngeal intubation.

### 2.2. Description of the Surgical Procedure

#### 2.2.1. Step 1. Harvesting the Graft

**Figure 2:**
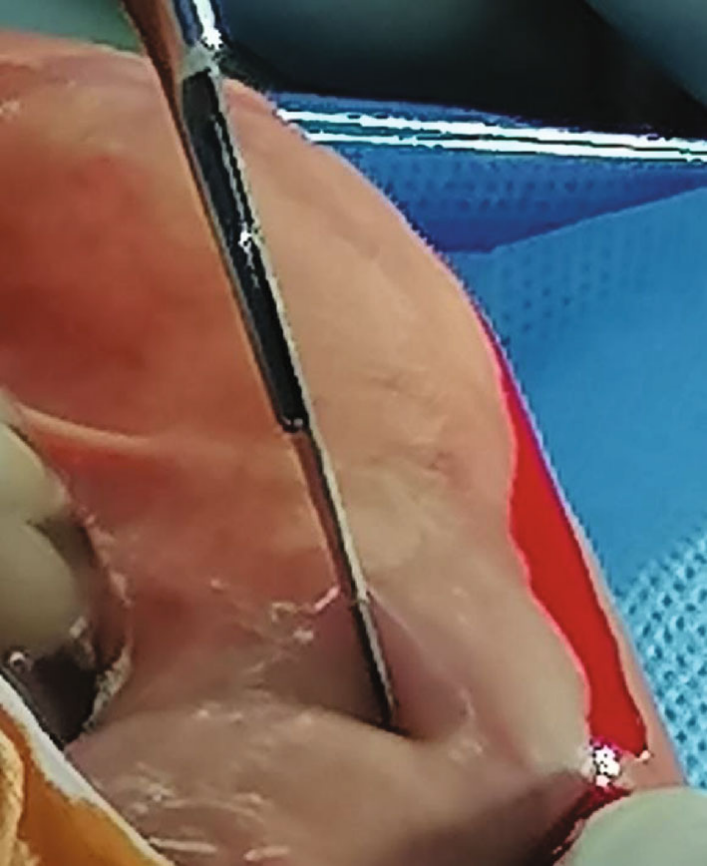
Harvesting oral mucosa graft from the lower lip, and a wheal raised with epinephrine solution.

**Figure 3:**
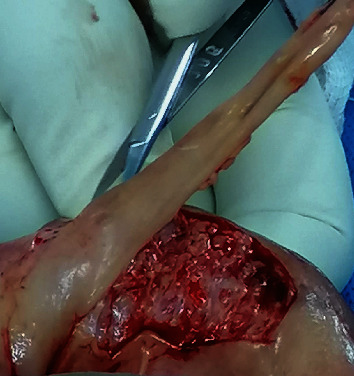
Oral mucosa graft being harvested from the lower lip.

A 7 cm × 2 cm lower lip oral mucosa graft starting from the right lateral angle of the mouth extending to the left cheek (Figures 2 and 3) was harvested. The submucosa was injected with lidocaine mixed with epinephrine (1 : 200,000) to raise a wheal which helped with easy harvesting of the graft while ensuring adequate haemostasis. A gauze was placed appropriately in the pharynx to prevent blood from entering the trachea or oesophagus. The mucosal defect at the donor site was left to heal by secondary intention. The graft was defatted removing all muscle fibers to retain only mucosa and lamina propria. Tiny fenestrations were made into it and then kept in saline until needed.

#### 2.2.2. Step 2. Preparation of the Recipient Site

A midline perineal incision was made through the skin, subcutaneous tissues, and fascia. The perineal muscles were divided in the midline and carefully dissected away from the urethra. The urethra was then circumferentially mobilized off the corporeal bodies from the bulbar to the penile urethra going beyond the strictured segments as defined by a catheter passed per the urethral meatus and a bougie dilator proximally from the cystostomy wound by sharp dissection with fine scissors.

**Figure 4:**
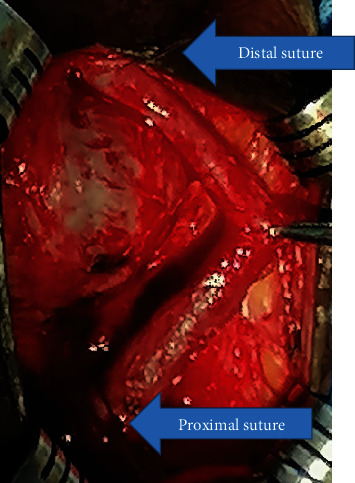
Urethra mobilized completely off the ventral surface of the corporeal bodies and rotated 180° with the aid of distal and proximal sutures (arrows).

**Figure 5:**
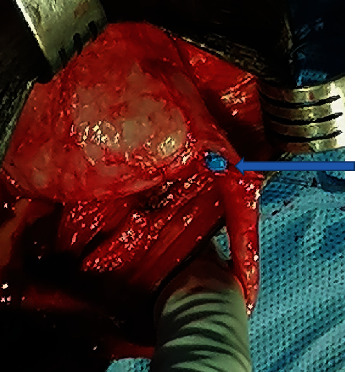
Initial dorsal incision on the catheter tip (arrow) to expose a healthy urethral lumen.

**Figure 6:**
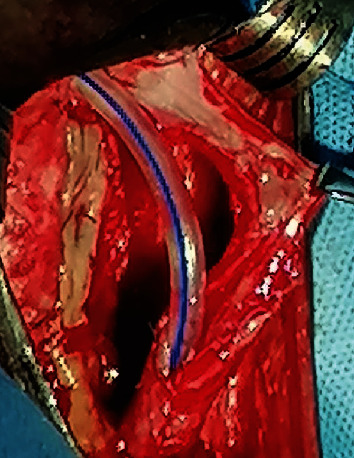
The narrowed urethral segment has been opened up under direct vision, and the oral mucosa graft has been quilted to the underside of the corporeal bodies.

The urethra was then rotated 180° bringing the dorsal aspect ventrally with the aid of sutures placed proximally and distally on the dorsal aspect of the urethra (Figure 4). A dorsal stricturotomy was made over the strictured segment starting distally from the immediately adjacent patent urethral segment over the catheter tip (Figures 5 and 6). This was to ensure that the urethral mucosa was visible throughout the stricturotomy.

**Figure 7:**
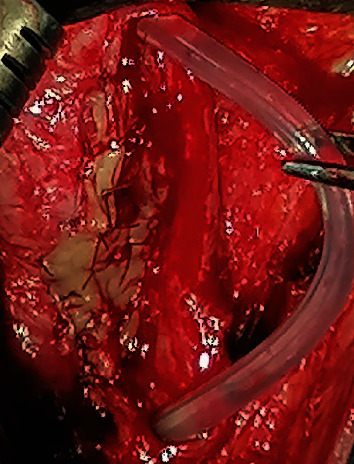
Suturing the left edge to the urethral plate to the left lateral aspect of the graft.

The oral mucosa graft was quilted to the ventral aspect of the corporeal bodies, and the left edge of the urethral plate was sutured to the left lateral aspect of the graft in interrupted fashion using vicryl 4/0 (Figures 6 and 7).

**Figure 8:**
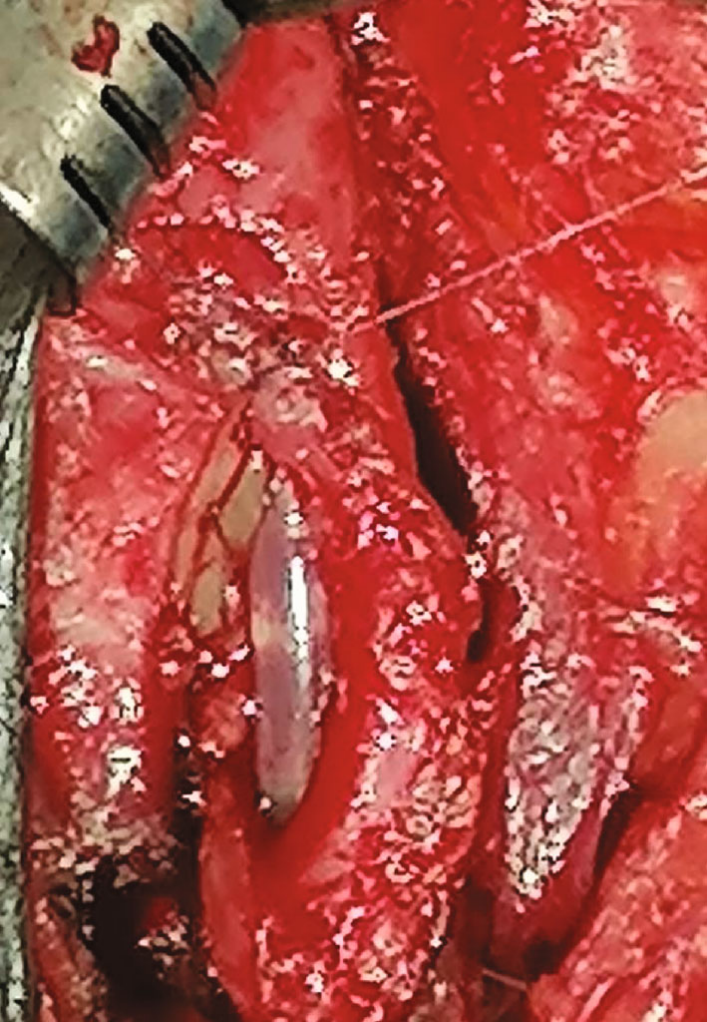
Suturing the right edge of the graft to the right aspect of the urethral plate over the size 16Fr catheter.

A size of 16Fr urethral catheter was advanced into the bladder, and the contralateral edge of the urethral plate was then sutured to the right edge of the oral mucosa graft over it (Figure 8).

### 2.3. Postoperative Management

**Figure 9:**
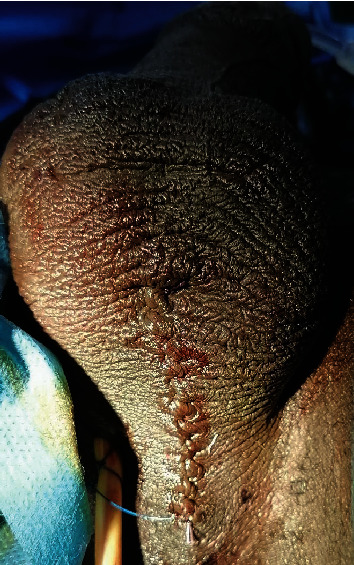
Final wound closure with a catheter drain left in situ.

Postoperatively, a size of 16Fr catheter wound drain was left in situ and closed wound dressing done (Figure 9). A spigot was applied to the urethral catheter which was then plastered loosely to the thigh to prevent excessive movement within the urethra. A urine bag was connected to the suprapubic catheter for continuous drainage of urine. Patient was continued on parenteral cefuroxime for one more week. The wound drain was removed on day 2 when it was found not to drain any longer.

**Figure 10:**
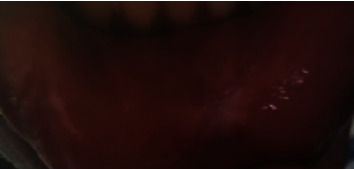
Healed donor site.

The donor site healed within 7 days without any complications with no special dressing (Figure 10).

**Figure 11:**
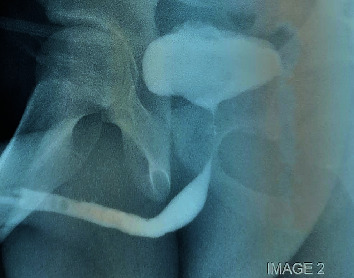
RUG in the left oblique view 4 weeks posturethroplasty showing normal caliber anterior urethra with no contrast extravasation.

Both the suprapubic and urethral catheters were removed 4 weeks postoperatively when RUG showed no urinary extravasation (Figure 11). Patient has been reviewed at 3, 6, and 12 months postoperatively with no urinary concerns.

## 3. Discussion

Poor quality latex catheters are known to cause extensive anterior urethral strictures mainly from allergic reactions and urethritis [13–15].

Repeated minimally invasive treatment options like dilatation and direct vision internal urethrotomy (DVIU) have not been found useful in the management of complex anterior strictures [16] as happened in this patient.

The management of such complex strictures relies on tissue transfer techniques or staged urethroplasty [17, 18].

Staged urethroplasty beyond the high revision rates has the tendency to increase morbidity and financial burden and has challenges with psychosocial adjustment [17, 19]. In a boy of school going age, a prompt solution in a single stage is a welcome relief as it allows him to return to normalcy without the need for regular hospital attendance.

The use of genital fasciocutaneous flaps is a viable option for managing such complex anterior urethral strictures in a single stage [9]. It also has a durable long-term success rate of about 79% [20]. However, it may be complicated by flap and proximal penile skin necrosis, penile oedema, wound haematoma, infection, etc. There is also the risk of penile shortening and chordee beyond issues of cosmesis [21–23].

Buccal mucosa has become the graft material of choice in the management of complex anterior urethral strictures. This is because it is readily available and easily harvested from the cheek or lip, allowing for the scar to be hidden with low oral morbidity. It is also hairless, with a thick elastin-rich epithelium, which makes it tough yet easy to handle and a thin and highly vascular lamina propria, which facilitates graft take and survival [24]. We chose the lower lip mucosa for ease of harvest and avoidance of injury to Stenson's duct.

The use of skin, bladder, lingual, and rectal mucosa for onlay urethroplasty has also been reported in literature when certain conditions preclude the use of buccal mucosa [25].

Buccal mucosa grafts for dorsal onlay urethroplasty as first described by Barbagli is a versatile technique as it obviates a lot of the challenges associated with the ventral onlay approach such as sacculations, diverticulum formation, and troublesome postvoid dribbling due to inadequacy of the graft support [26]. This is because the ventral aspect of the corporeal bodies provide a much more solid mechanical support for the graft compared with the corpus spongiosum [10].

However, the abundant corpus spongiosum of the proximal bulbar urethra lends adequate support to a ventrally placed graft as opposed to strictures involving the distal bulbar and penile urethra, in which the dorsal approach is preferable due to the paucity of the corpus spongiosum [27, 28].

The success rate of buccal mucosa graft urethroplasty has been estimated in literature to range from 80 to 95% with the dorsal onlay approach having a slight advantage over the ventral approach [29–31].

We chose the oral mucosa graft dorsal onlay urethroplasty technique for this patient because it is fairly simple to harvest the graft with little morbidity at the donor site and has excellent cosmesis with durable long-term success rate.

This patient had no major complications following the surgery and has a good stream of urine with a peak flow rate of 24mls/s after one year of follow-up.

## 4. Conclusion

The oral mucosa graft dorsal onlay urethroplasty for complex anterior urethral strictures is applicable to complex anterior urethral strictures in children. The technique is simple to perform with minimal morbidities and has good outcomes.

## Data Availability

The data on this case including radiograms are available for review upon request.
